# 2,4-Dimethyl­anilinium perchlorate

**DOI:** 10.1107/S1600536810017253

**Published:** 2010-06-05

**Authors:** Wen-Xian Liang

**Affiliations:** a Ordered Matter Science Research Center, College of Chemistry and Chemical Engineering, Southeast University, Nanjing 210096, People’s Republic of China

## Abstract

The crystal packing of the title compound, C_8_H_12_N^+^·ClO_4_^−^, is stabilized by N—H⋯O hydrogen bonds, the protonated amine group acting as a hydrogen-bond donor with the perchlorate O atoms as acceptors. These connect neighbouring cations and anions, forming a two-dimensional network. Variable-temperature dielectric constant measurements on the salt indicated that no distinct phase transition occurred within the measured temperature range of 80–293 K.

## Related literature

For the synthesis and characterization of 2,4-dimethyl­anilinium phosphate, see: Fábry *et al.* (2001 ▶). For the structure of 2,4,6-trimethyl­anilinium iodide, see: Lemmerer & Billing (2007 ▶).
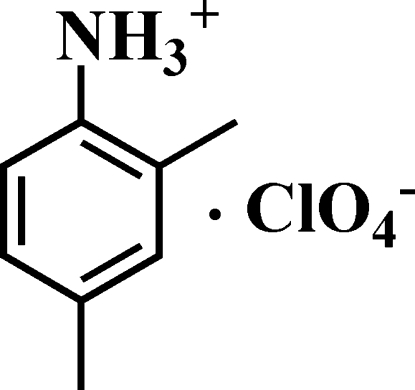


## Experimental

### 

#### Crystal data

C_8_H_12_N^+^·ClO_4_^−^
*M* *_r_* = 221.64
Monoclinic, 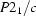

*a* = 9.3299 (19) Å
*b* = 7.1947 (14) Å
*c* = 15.176 (3) Å
β = 97.43 (3)°
*V* = 1010.2 (3) Å^3^
*Z* = 4
Mo *K*α radiation
μ = 0.37 mm^−1^
*T* = 293 K
0.45 × 0.30 × 0.15 mm

#### Data collection

Rigaku SCXmini diffractometer
Absorption correction: multi-scan (*CrystalClear*; Rigaku, 2005 ▶) *T*_min_ = 0.884, *T*_max_ = 0.950
9986 measured reflections
2318 independent reflections
1970 reflections with *I* > 2σ(*I*)
*R*_int_ = 0.030

#### Refinement

*R*[*F* ^2^ > 2σ(*F* ^2^)] = 0.044
*wR*(*F* ^2^) = 0.122
*S* = 1.09
2318 reflections
130 parameters
H-atom parameters constrained
Δρ_max_ = 0.22 e Å^−3^
Δρ_min_ = −0.38 e Å^−3^

### 

Data collection: *CrystalClear* (Rigaku, 2005 ▶); cell refinement: *CrystalClear* data reduction: *CrystalClear*; program(s) used to solve structure: *SHELXS97* (Sheldrick, 2008 ▶); program(s) used to refine structure: *SHELXL97* (Sheldrick, 2008 ▶); molecular graphics: *SHELXTL* (Sheldrick, 2008 ▶); software used to prepare material for publication: *PRPKAPPA* (Ferguson, 1999 ▶).

## Supplementary Material

Crystal structure: contains datablocks I, global. DOI: 10.1107/S1600536810017253/sj2798sup1.cif

Structure factors: contains datablocks I. DOI: 10.1107/S1600536810017253/sj2798Isup2.hkl

Additional supplementary materials:  crystallographic information; 3D view; checkCIF report

## Figures and Tables

**Table 1:** Hydrogen-bond geometry (Å, °)

*D*—H⋯*A*	*D*—H	H⋯*A*	*D*⋯*A*	*D*—H⋯*A*
N1—H1*B*⋯O1^i^	0.89	2.24	3.002 (3)	143
N1—H1*B*⋯O4^i^	0.89	2.53	3.236 (3)	137
N1—H1*A*⋯O2^ii^	0.89	2.16	2.983 (3)	153
N1—H1*C*⋯O3	0.89	2.15	2.994 (3)	159

Symmetry codes: (i) 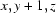; (ii) 
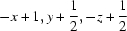
.

**Fig. 1.**
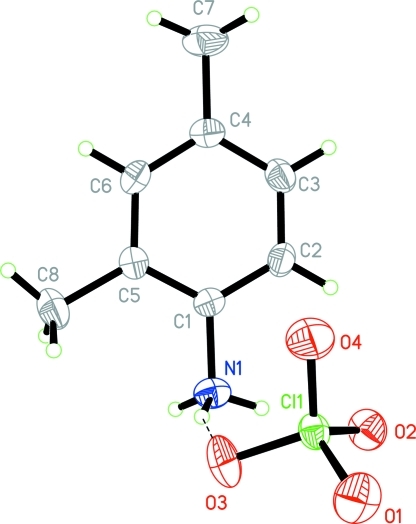
The asymmetric unit of the title compound, with the displacement ellipsoids were drawn at the 30% probability level. A hydrogen bond is shown as a dashed line.

**Fig. 2.**
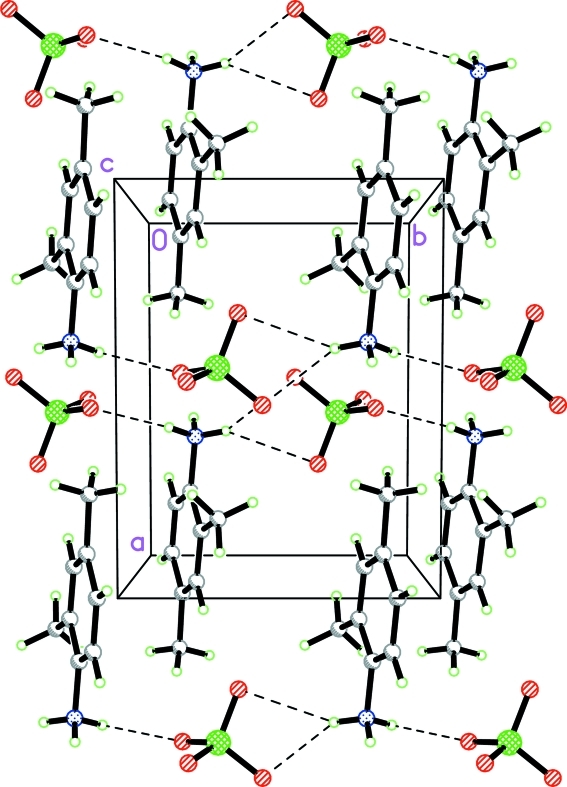
Packing diagram of the title compound, showing the structure along the a axis. Hydrogen bonds are shown as dashed lines.

## supplementary crystallographic information

### Comment

Recently, Fábry *et al.* (2001) reported the synthesis and characterization of the 2,4-dimethylanilinium phosphate. Lemmerer & Billing (2007) researched the crystal structure of the 2,4,6-trimethylanilinium iodide. This paper reports the crystal structure and dielectric properties of the related salt 2,4-dimethylanilinium perchlorate. The asymmetric unit of title compound, C_8_H_12_N^+^.ClO_4_^-^, contains a 2,4-dimethylanilinium cation and one perchlorate anion (Fig.1).  The ammonium cations stack head-to-tail with no π-π interactions. The crystal packing is stabilized by N—H···O hydrogen bonds, the protonated amine group acting as a hydrogen-bond donor with the perchlorate O atoms as acceptors. These connect neighbouring cations and anions to form a two-dimensional network (Fig.2). In addition, the dielectric constant of title compound as a function of temperature indicates that the permittivity is basically temperature-independent (dielectric constant equaling to 2.6 to 4.5) from 80k to 293k, suggesting that no distinct phase transition occurred within the measured temperature range.

### Experimental

2,4-dimethylbenzenamine (1.21 g, 10 mmol) and perchloric acid (1 g, 10 mmol) were mixed and the 2,4-dimethylbenzenamine perchlorate was obtained, then it was dissolved in water (3 ml), ethanol (20 ml), and the solution was filtered. After slowly evaporating over a period of 3 d, colorless prism crystals of the title compound suitable for diffraction were isolated. CAUTION:  Although no problems were encountered in this work, perchlorate compounds are potentially explosive.  They should be prepared in small amounts and handled with care.

### Refinement

Positional parameters of all the H atoms were calculated geometrically and were allowed to ride on the C, N atoms to which they are bonded, with C—H = 0.93 to 0.97 Å, U_iso_(H) = 1.2 Ueq(C), N—H = 0.89 Å, U_iso_(H)= 1.5 U_eq_(N).

### Crystal data

**Table:**

C_8_H_12_N^+^·ClO_4_^−^	*F*(000) = 464
*M**_r_* = 221.64	*D*_x_ = 1.457 Mg m^−^^3^
Monoclinic, *P*2_1_/*c*	Mo *K*α radiation, λ = 0.71073 Å
Hall symbol: -P 2ybc	Cell parameters from 1970 reflections
*a* = 9.3299 (19) Å	θ = 3.1–27.5°
*b* = 7.1947 (14) Å	µ = 0.37 mm^−^^1^
*c* = 15.176 (3) Å	*T* = 293 K
β = 97.43 (3)°	Prism, colorless
*V* = 1010.2 (3) Å^3^	0.45 × 0.30 × 0.15 mm
*Z* = 4	

### Data collection

**Table:**

Rigaku SCXmini diffractometer	2318 independent reflections
Radiation source: fine-focus sealed tube	1970 reflections with *I* > 2σ(*I*)
graphite	*R*_int_ = 0.030
Detector resolution: 13.6612 pixels mm^-1^	θ_max_ = 27.5°, θ_min_ = 3.1°
CCD_Profile_fitting scans	*h* = −12→12
Absorption correction: multi-scan (*CrystalClear*; Rigaku, 2005)	*k* = −9→9
*T*_min_ = 0.884, *T*_max_ = 0.950	*l* = −19→19
9986 measured reflections	

### Refinement

**Table:**

Refinement on *F*^2^	Secondary atom site location: difference Fourier map
Least-squares matrix: full	Hydrogen site location: inferred from neighbouring sites
*R*[*F*^2^ > 2σ(*F*^2^)] = 0.044	H-atom parameters constrained
*wR*(*F*^2^) = 0.122	*w* = 1/[σ^2^(*F*_o_^2^) + (0.0568*P*)^2^ + 0.4369*P*] where *P* = (*F*_o_^2^ + 2*F*_c_^2^)/3
*S* = 1.09	(Δ/σ)_max_ < 0.001
2318 reflections	Δρ_max_ = 0.22 e Å^−^^3^
130 parameters	Δρ_min_ = −0.38 e Å^−^^3^
0 restraints	Extinction correction: *SHELXL97* (Sheldrick, 2008)
Primary atom site location: structure-invariant direct methods	Extinction coefficient: 0.0014 (1)

### Special details

**Table:**

Geometry. All esds (except the esd in the dihedral angle between two l.s. planes) are estimated using the full covariance matrix. The cell esds are taken into account individually in the estimation of esds in distances, angles and torsion angles; correlations between esds in cell parameters are only used when they are defined by crystal symmetry. An approximate (isotropic) treatment of cell esds is used for estimating esds involving l.s. planes.
Refinement. Refinement of *F*^2^ against ALL reflections. The weighted *R*-factor wR and goodness of fit *S* are based on *F*^2^, conventional *R*-factors *R* are based on *F*, with *F* set to zero for negative *F*^2^. The threshold expression of *F*^2^ > σ(*F*^2^) is used only for calculating *R*-factors(gt) etc. and is not relevant to the choice of reflections for refinement. *R*-factors based on *F*^2^ are statistically about twice as large as those based on *F*, and *R*- factors based on ALL data will be even larger.

### Fractional atomic coordinates and isotropic or equivalent isotropic displacement parameters (Å^2^)

**Table:**

	*x*	*y*	*z*	*U*_iso_*/*U*_eq_	
Cl1	0.44022 (5)	0.20914 (6)	0.11652 (3)	0.03993 (17)	
N1	0.37293 (19)	0.7004 (2)	0.12219 (13)	0.0489 (5)	
H1A	0.4247	0.6911	0.1755	0.073*	
H1B	0.3888	0.8107	0.0986	0.073*	
H1C	0.3987	0.6108	0.0870	0.073*	
C5	0.1146 (2)	0.7244 (3)	0.05939 (13)	0.0400 (4)	
O2	0.47637 (18)	0.3056 (2)	0.19934 (10)	0.0576 (4)	
C6	−0.0291 (2)	0.7058 (3)	0.07285 (14)	0.0453 (5)	
H6	−0.1004	0.7328	0.0259	0.054*	
O3	0.4530 (2)	0.3369 (2)	0.04594 (11)	0.0687 (5)	
C1	0.2174 (2)	0.6817 (2)	0.13112 (13)	0.0374 (4)	
C3	0.0362 (2)	0.6056 (3)	0.22160 (14)	0.0508 (5)	
H3	0.0108	0.5647	0.2756	0.061*	
C2	0.1796 (2)	0.6221 (3)	0.21116 (14)	0.0489 (5)	
H2	0.2508	0.5931	0.2579	0.059*	
O1	0.5376 (2)	0.0565 (2)	0.11199 (14)	0.0717 (5)	
C4	−0.0713 (2)	0.6491 (3)	0.15282 (14)	0.0447 (5)	
O4	0.29667 (19)	0.1392 (3)	0.11030 (13)	0.0764 (6)	
C8	0.1543 (3)	0.7883 (4)	−0.02867 (16)	0.0648 (7)	
H8A	0.0687	0.7957	−0.0711	0.097*	
H8B	0.2206	0.7014	−0.0493	0.097*	
H8C	0.1989	0.9086	−0.0219	0.097*	
C7	−0.2294 (3)	0.6367 (5)	0.1648 (2)	0.0716 (8)	
H7A	−0.2563	0.7443	0.1962	0.107*	
H7B	−0.2455	0.5269	0.1981	0.107*	
H7C	−0.2869	0.6309	0.1076	0.107*	

### Atomic displacement parameters (Å^2^)

**Table:**

	*U*^11^	*U*^22^	*U*^33^	*U*^12^	*U*^13^	*U*^23^
Cl1	0.0472 (3)	0.0343 (3)	0.0378 (3)	−0.00671 (19)	0.00362 (19)	0.00247 (18)
N1	0.0431 (10)	0.0372 (9)	0.0668 (12)	−0.0048 (7)	0.0083 (8)	−0.0034 (8)
C5	0.0499 (11)	0.0324 (9)	0.0376 (10)	−0.0001 (8)	0.0056 (8)	−0.0034 (8)
O2	0.0645 (10)	0.0629 (11)	0.0441 (9)	−0.0085 (8)	0.0022 (7)	−0.0104 (7)
C6	0.0447 (11)	0.0460 (11)	0.0425 (11)	0.0061 (9)	−0.0051 (8)	−0.0041 (9)
O3	0.1083 (15)	0.0501 (9)	0.0500 (9)	−0.0033 (10)	0.0185 (9)	0.0161 (8)
C1	0.0360 (9)	0.0281 (9)	0.0475 (11)	−0.0009 (7)	0.0035 (8)	−0.0033 (8)
C3	0.0580 (13)	0.0563 (14)	0.0386 (10)	−0.0109 (11)	0.0086 (9)	0.0008 (10)
C2	0.0499 (12)	0.0486 (12)	0.0449 (11)	−0.0038 (10)	−0.0067 (9)	0.0094 (10)
O1	0.0846 (13)	0.0446 (9)	0.0855 (13)	0.0141 (9)	0.0097 (10)	−0.0015 (9)
C4	0.0407 (10)	0.0450 (11)	0.0490 (11)	−0.0020 (9)	0.0087 (8)	−0.0127 (10)
O4	0.0571 (11)	0.0905 (14)	0.0783 (12)	−0.0318 (10)	−0.0041 (9)	−0.0005 (11)
C8	0.0829 (18)	0.0708 (17)	0.0423 (12)	−0.0117 (14)	0.0141 (11)	0.0031 (12)
C7	0.0469 (13)	0.090 (2)	0.0805 (18)	−0.0010 (14)	0.0197 (12)	−0.0212 (16)

### Geometric parameters (Å, °)

**Table:**

Cl1—O4	1.4225 (17)	C1—C2	1.377 (3)
Cl1—O3	1.4281 (16)	C3—C2	1.373 (3)
Cl1—O1	1.4326 (18)	C3—C4	1.386 (3)
Cl1—O2	1.4372 (16)	C3—H3	0.9300
N1—C1	1.481 (2)	C2—H2	0.9300
N1—H1A	0.8900	C4—C7	1.512 (3)
N1—H1B	0.8900	C8—H8A	0.9600
N1—H1C	0.8900	C8—H8B	0.9600
C5—C6	1.388 (3)	C8—H8C	0.9600
C5—C1	1.389 (3)	C7—H7A	0.9600
C5—C8	1.504 (3)	C7—H7B	0.9600
C6—C4	1.385 (3)	C7—H7C	0.9600
C6—H6	0.9300		
			
O4—Cl1—O3	110.33 (13)	C2—C3—C4	121.0 (2)
O4—Cl1—O1	108.85 (13)	C2—C3—H3	119.5
O3—Cl1—O1	110.01 (12)	C4—C3—H3	119.5
O4—Cl1—O2	109.96 (11)	C3—C2—C1	119.57 (19)
O3—Cl1—O2	108.19 (11)	C3—C2—H2	120.2
O1—Cl1—O2	109.48 (11)	C1—C2—H2	120.2
C1—N1—H1A	109.5	C6—C4—C3	117.8 (2)
C1—N1—H1B	109.5	C6—C4—C7	121.0 (2)
H1A—N1—H1B	109.5	C3—C4—C7	121.2 (2)
C1—N1—H1C	109.5	C5—C8—H8A	109.5
H1A—N1—H1C	109.5	C5—C8—H8B	109.5
H1B—N1—H1C	109.5	H8A—C8—H8B	109.5
C6—C5—C1	116.46 (18)	C5—C8—H8C	109.5
C6—C5—C8	120.9 (2)	H8A—C8—H8C	109.5
C1—C5—C8	122.6 (2)	H8B—C8—H8C	109.5
C4—C6—C5	123.14 (19)	C4—C7—H7A	109.5
C4—C6—H6	118.4	C4—C7—H7B	109.5
C5—C6—H6	118.4	H7A—C7—H7B	109.5
C2—C1—C5	122.05 (19)	C4—C7—H7C	109.5
C2—C1—N1	118.36 (18)	H7A—C7—H7C	109.5
C5—C1—N1	119.58 (18)	H7B—C7—H7C	109.5

### Hydrogen-bond geometry (Å, °)

**Table:**

*D*—H···*A*	*D*—H	H···*A*	*D*···*A*	*D*—H···*A*
N1—H1B···O1^i^	0.89	2.24	3.002 (3)	143
N1—H1B···O4^i^	0.89	2.53	3.236 (3)	137
N1—H1A···O2^ii^	0.89	2.16	2.983 (3)	153
N1—H1C···O3	0.89	2.15	2.994 (3)	159

Symmetry codes: (i) *x*, *y*+1, *z*; (ii) −*x*+1, *y*+1/2, −*z*+1/2.
